# 267. Therapeutic Management of Bacterial Brain Abscess: An Overview of Diagnosis and Outcomes

**DOI:** 10.1093/ofid/ofab466.469

**Published:** 2021-12-04

**Authors:** Cristina G Corsini Campioli, John Raymond U Go, John C O’Horo, M Rizwan Sohail, M Rizwan Sohail

**Affiliations:** 1 Mayo Clinic, Rochester, MN; 2 Mayo Clinic Rochester, Rochester, MN; 3 Baylor College of Medicine, Sugarland, TX

## Abstract

**Background:**

We describe and compare the clinical, diagnostic evaluation and outcomes of patients who underwent therapeutic management for pyogenic brain abscess.

**Methods:**

We retrospectively reviewed adults who presented with pyogenic brain abscess from January 1, 2009 through June 30, 2020.

**Results:**

231 patients were identified during the study period. Sixty-one (26.4%) patients received antibiotic therapy alone, and 170 (73.6%) had a combination of antibiotic therapy and surgical intervention. The median age for the medical and combined therapy group was 59 years and 58 years, respectively. Patients who received medical treatment had a higher prevalence of infective endocarditis than those who received combined therapy (6.6% vs. 0.6%; P=0.005). The medical therapy group was more likely to have brain MRI and cranial CT than the patients with combined therapy (75.4% vs. 63.5%; P=0.041). Midline shift (11.5% vs. 31.2%; P=0.002), a single (21% vs. 83%; P=0.001) and greater size (1.4 cm vs. 2.5 cm; P=0.007) brain abscess was significant when comparing medical vs. surgically managed abscess. Stereotactic surgical technique was the preferred diagnostic approach for the medical group (65.6% vs. 46.5%; P=0.010), and excision/craniotomy for the combined group (31.1% vs. 53.5%; P=0.002). Streptococcus viridans group was the predominant organism (32.8% and 25.9%; P=0.30). Compared to those who received combined therapy, patients with medical therapy alone were most likely to receive cephalosporin (72.1% vs. 41.2%; P=< 0.0001), vancomycin (23% vs. 12.4%; P=0.047) and metronidazole (27.9% vs. 14.7%; P=0.022). In both groups, median duration of antimicrobial therapy was 42 days (P=0.12). Patients with medical therapy alone had a higher mortality rate (18% vs. 7.1%; p=0.014) but less neurologic sequelae (21.3% vs. 30.6%; P=0.16) compared with combined therapy.

Medical Management. Organism isolated in the medical management group

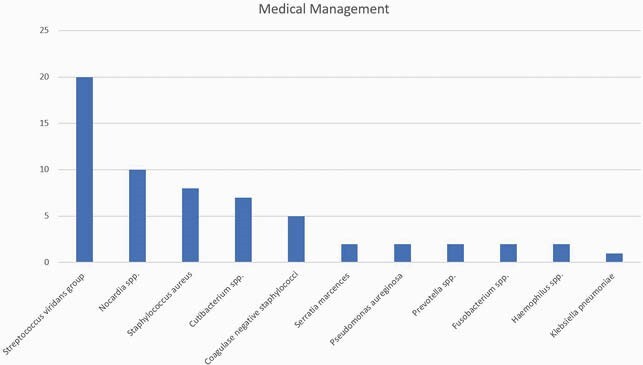

Combined Management. Organism isolated in the combined management group

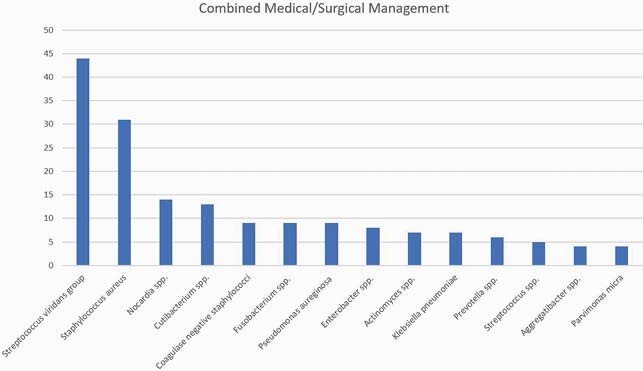

Demographic and Clinical Characteristics of Patients with Brain Abscess who Underwent Therapeutic Management

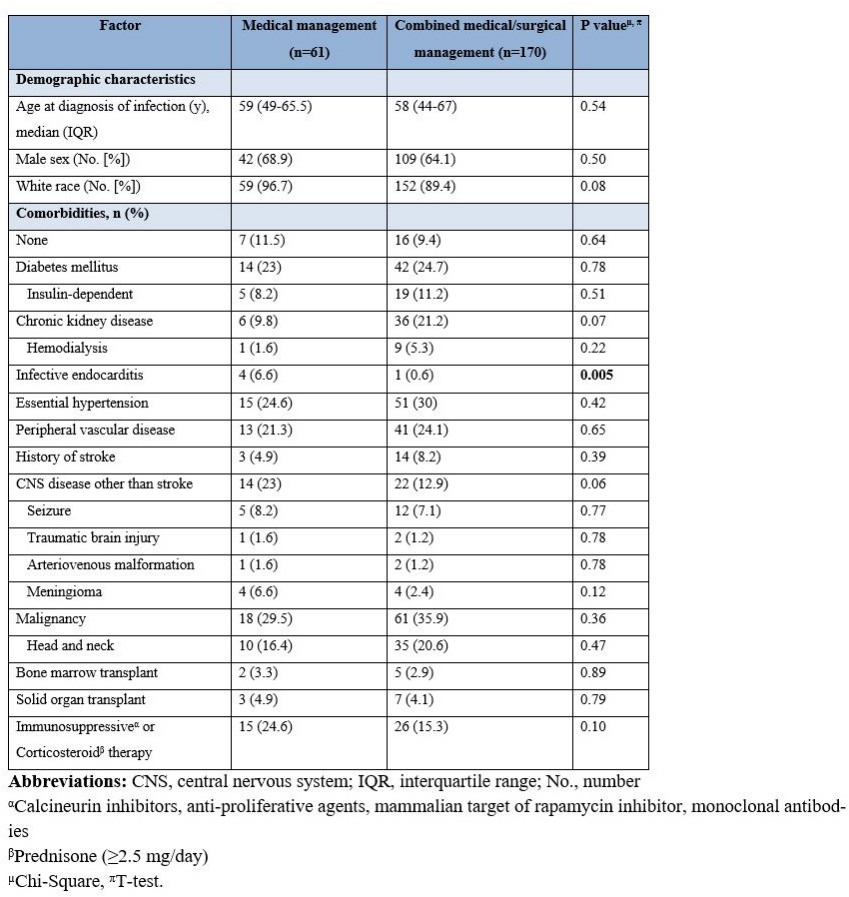

**Conclusion:**

Most patients with pyogenic brain abscess had no identified risk factors, and brain MRI and cranial CT were the diagnostic imaging modalities of choice. Compared to those who received medical therapy alone, patients with combined treatment had a single and greater size fluid collection with the presence of midline shift. A prompt combined surgical and medical approach with prolonged antimicrobial therapy can cure the infection.

Outcomes of Patients with Bacterial Brain Abscess

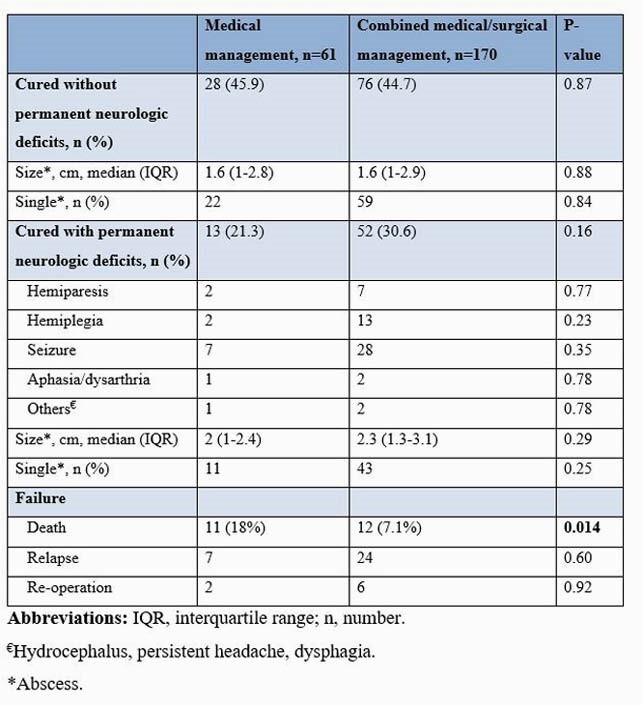

Radiologic and Surgical Diagnosis of Patient with Brain Abscess who Underwent Therapeutic Management

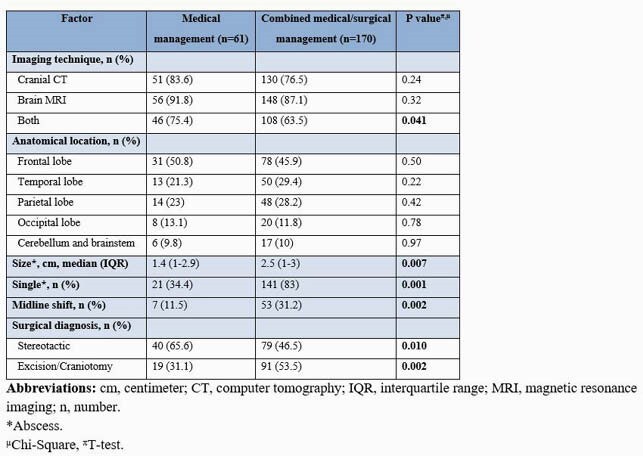

**Disclosures:**

**John C. O’Horo, Sr., MD, MPH**, **Bates College and Elsevier Inc** (Consultant) **M. Rizwan Sohail, MD**, **Medtronic Inc., Philips, and Aziyo Biologics, Inc** (Consultant) **M. Rizwan Sohail, MD**, Aziyo Biologics (Individual(s) Involved: Self): Consultant; Philips (Individual(s) Involved: Self): Consultant

